# Supplementary material for: Analyses of Transcriptomics upon IL-1β-Stimulated Mouse Chondrocytes and the Protective Effect of Catalpol through the NOD2/NF-κB/MAPK Signaling Pathway
Source: Molecules. 2023 Feb 7;28(4):1606. doi: 10.3390/molecules28041606 (PMC9962284; doi:10.3390/molecules28041606)
Supplement: Supplementary file 1 [file molecules-28-01606-s001.zip › raw data/KEGG/IL-1b_vs_N/Enrich_plot/IL-1b_vs_N.DEG_down_KEGG_enriched.pdf]

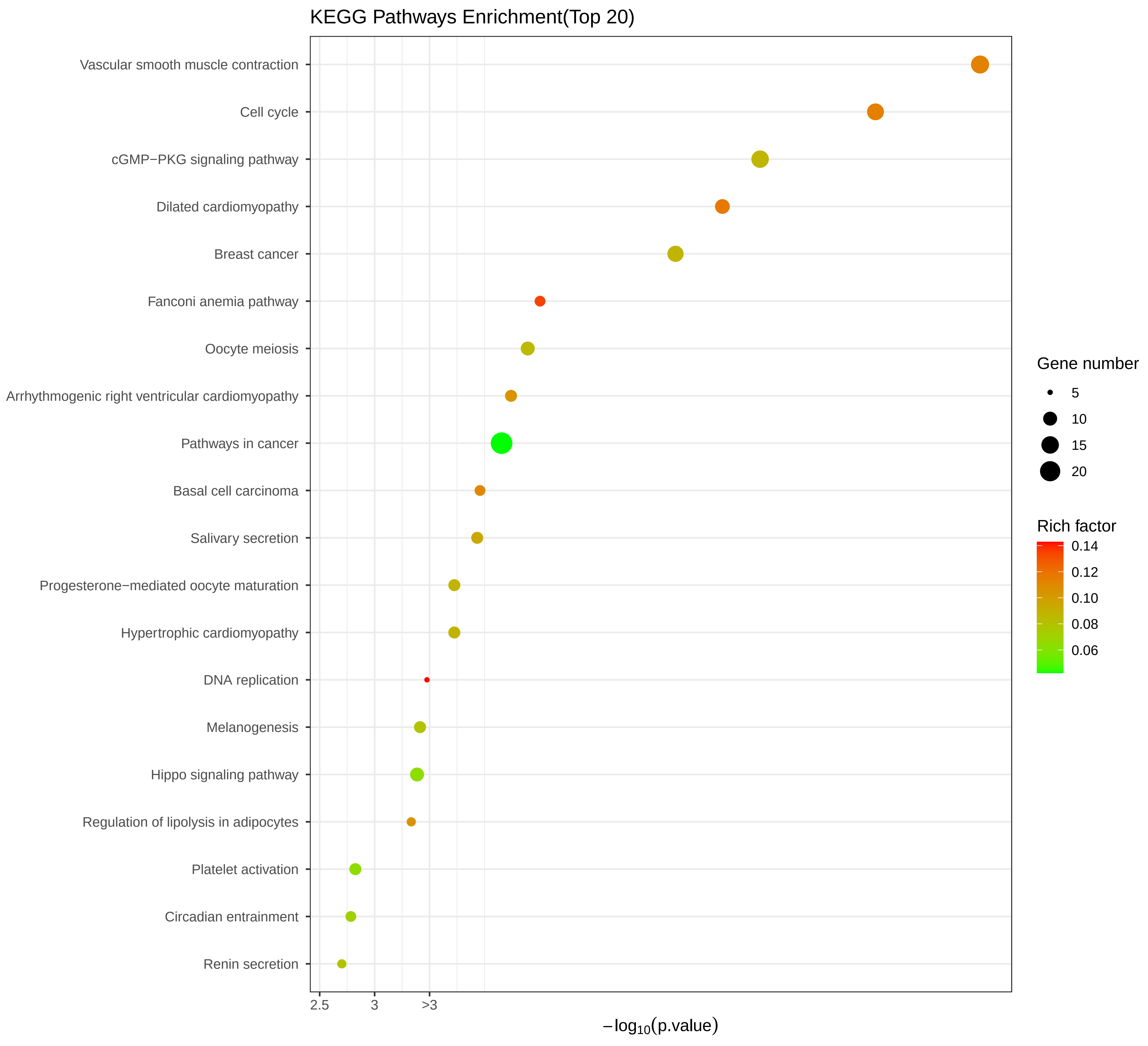

KEGG Pathways Enrichment(Top 20)
Vascular smooth muscle contraction
Cell cycle
cGMP−PKG signaling pathway
Dilated cardiomyopathy
Breast cancer
Fanconi anemia pathway
Oocyte meiosis
Arrhythmogenic right ventricular cardiomyopathy
Pathways in cancer
Basal cell carcinoma
Salivary secretion
Progesterone−mediated oocyte maturation
Hypertrophic cardiomyopathy
DNA replication
Melanogenesis
Hippo signaling pathway
Regulation of lipolysis in adipocytes
Platelet activation
Circadian entrainment
Renin secretion
2.5
3
>3
$-\log_{10}(p.value)$
Gene number
5
10
15
20
Rich factor
0.14
0.12
0.10
0.08
0.06
